# Essential and core books for veterinary medicine

**DOI:** 10.5195/jmla.2018.391

**Published:** 2018-07-01

**Authors:** Heather K. Moberly, Jessica R. Page

**Affiliations:** Dorothy G. Whitley Professor and Coordinator of Veterinary Services, Medical Sciences Library, University Libraries, Texas A&M University, College Station, TX; Assistant Professor and Head, Hodesson Veterinary Medicine Library, University Libraries, The Ohio State University, 225 Veterinary Medicine Academic Building, Columbus, OH 43210

## Abstract

**Objectives:**

This study defined core and essential lists of recent, English-language veterinary medicine books using a data-driven methodology for potential use by a broad audience, including libraries that are building collections supporting veterinary sciences and One Health initiatives.

**Methods:**

Book titles were collected from monograph citation databases, veterinary examination reading lists, veterinary college textbook and library reserve lists, and published bibliographies. These lists were combined into a single list with titles ranked by the number of occurrences.

**Results:**

The methodology produced a core list of 122 monographs and an essential list of 33 titles. All titles are recent, edition neutral, English language monographs. One title is out of print.

**Conclusions:**

The methodology captured qualitative and quantitative input from four distinct populations who use veterinary monographs: veterinary practitioners, educators, researchers, and librarians. Data were collected and compiled to determine core and essential lists that represented all groups. Unfortunately, data are not available for all subareas of veterinary medicine, resulting in uneven subject coverage. This methodology can be replicated and adapted for other subject areas.

## INTRODUCTION

Veterinary practitioners have consistently been shown to be a profession of readers [1–5]. Books were the top information source for consultation by veterinarians at their practices in 1978 [6] and remained a main source of information, in addition to Internet sources, in 2000 [3]. By 2015, books were the second main source, after consulting with colleagues, for seeking information about a difficult case [5].

Academic, research, and clinical libraries use core resource lists to benchmark against other specialist collections, support selective collection development, and guide choices for specialty situations. Lists of core resources not only help guide selections for home and practice libraries, but also capture knowledge and provide a foundation for benchmarking and collection development in information specialties, such as veterinary medicine librarianship, that are experiencing both expertise loss through retirement and shifts in responsibilities [7] and increasing interest in the literature due to new veterinary schools and pre-veterinary and technical undergraduate curricula. However, in this era of evidence-based initiatives that encourage integrating human, animal, and environmental health, known as One Health [8], interest in veterinary literature also comes from curricula of human medical schools, public health and comparative medicine programs, and veterinary and human medical practitioners. In human medicine, which has a strong history of core lists, lists have been generated from expert individuals [9, 10] or consensus [11] or through data-driven approaches [12].

Veterinary medicine collection development has previously been described holistically [13] and includes disciplines ranging from teaching and clinical work to research in basic, clinical, and applied sciences. Veterinary medicine is interdisciplinary, and core titles can include those from basic science and clinical veterinary medicine as well as human medicine, comparative medicine, agricultural sciences, and environmental sciences. The US national libraries acknowledge this interdisciplinary nature through a joint collection development policy covering veterinary medicine and related sciences to guide collaborative efforts. No one national library has responsibility for veterinary medicine collections, services, or indexing [14].

Medical core book lists have historically lacked a veterinary medicine section. This can be problematic because the interdisciplinary nature of medicine dictates that medical libraries often have some veterinary titles. A 1969 article described the need for medical school libraries to collect in veterinary medicine, in part to support laboratory animal and comparative medicine [15]. The need for a core list of veterinary titles was supported by a 1984 survey that found that medical school libraries held an average of 171 veterinary books and that 2 medical schools had developed core lists of veterinary medicine books by “adapting those of veterinary school libraries” [16]. However, the “Brandon/Hill Selected List of Books and Journals for the Small Medical Library,” published from 1965 to 2001 in the *Bulletin of the Medical Library Association,* did not include veterinary titles [17]. Also, Brandon and Hill published the “Selected List of Books and Journals in Allied Health Sciences” from 1984 to 2003 in the *Bulletin of the Medical Library Association* and *Journal of the Medical Library Association,* but this list did not include veterinary medicine because the programs were not accredited by the Committee on Allied Health Education and Accreditation of the American Medical Association, and the authors considered it an autonomous profession [18].

More recently, perhaps because of One Health, some newer core medical title lists have incorporated veterinary sections. Doody’s Core Titles, available by subscription, began publishing core lists of titles in 1993 for a number of health sciences specialties and disciplines, including veterinary medicine [19–21]. In 2011, *The Medical Library Association’s Master Guide to Authoritative Information Resources in the Health Sciences* was intended to offer an updated alternative for the Brandon/Hill lists and included a more thorough veterinary medicine list with fifteen subspecialties represented [9]. Veterinary librarians have written expert pieces about this topic that serve as foundational reading and information [22–25] and contribute to this study as source lists [9, 10, 12, 26].

Data-driven lists of core resources, which encompass more than one type of input, are less common. One data-driven veterinary monograph source list was identified and used a source list for the present study [12]. That list includes input from sources including “book reviews, subject bibliographies, recommended reading lists…and library catalogs, with the final list being determined by consulting veterinary faculty, graduate students and librarians.” That list, published in 2004, still holds value as a list that encompasses the broad aspects of veterinary medicine. However, in the present study, the authors intended to generate a leaner core list of titles using methods that were as objective as possible.

The most recent edition of the veterinary medicine core journal list is also data-driven and was compiled using a rubric based on four sets of objective and subjective data including indexing sources, journal ranking, expert librarian opinion, and inclusion on specialty examination reading lists [27, 28]. The goal of the present study was to develop a similar data-driven list of monographs by closely following that methodology despite differences in available data sources. However, largely due to the vast number of veterinary monographs as compared to veterinary serials and a lack of robust objective data, our study required developing a different methodology. In this mixed-methods study, we developed core and essential lists of veterinary medicine books from sources representing evidence from citation data and opinion from four populations: veterinary practitioners, academic researchers, educators, and librarians.

## METHODS

### Source data

The veterinary monographs included in this study cover veterinary medicine as broadly as possible. The sources of book titles—representing the input of veterinary practitioners, academic researchers, veterinary educators, and veterinary librarians—are summarized in Table 1.

**Table 1:** List of categories, the type of expert opinion they represent, and source material

Category	Primary constituents	Source material
Citation data	Academic researchers	Web of Science (WOS) Citation Database cited books BIOSIS Citation Index cited books Scopus cited books
American Veterinary Medical Association (AVMA) exam reading list data	Veterinary practitioners/specialists	Basic and Clinical Sciences Examination (BCSE) reading list Recognized Veterinary Specialty Organization reading lists (35 specialties)
Veterinary textbook lists and library reserve lists	Veterinary educators	Textbook lists and library reserves from 20 AVMA-accredited colleges of veterinary medicine
Published veterinary bibliographies	Veterinary librarians	4 veterinary librarian–authored bibliographies published within the previous 15 years

Book citation data, representing the input of researchers, was collected on October 10, 2016, from three citation databases. Due to the limited citation data for monographs, all available years of data were included from each source. The Web of Science (WOS) Core Collection (including article, book, and conference citation indexes across all disciplines) was searched across all years (1900–2016) for the subject “veterinary science” with document types limited to books and book chapters for titles that had been cited at least once [29]. The BIOSIS Citation Index was searched across all years (1926–2016) by topic containing “veterinary” with document types limited to books and book chapters for titles that had been cited at least once [30]. Scopus was searched across all years (1960–2016) by article title, abstract, or keywords containing “veterinary” with document types limited to books and book chapters for titles that had been cited at least once [31].

American Veterinary Medical Association (AVMA) exam reading list data represented the input of veterinary practitioners and specialists. These sources included the 2016 version of the Basic Clinical Sciences Examination (BCSE) reading list, downloaded from the AVMA website [32], and the Recognized Veterinary Specialty Organizations (RVSO) reading lists for 35 specialty examinations, downloaded from the Veterinary Specialty Boards Reading Lists Libraries Template [33] on October 12, 2016.

Veterinary textbook lists and library reserve lists, representing the input of veterinary educators, were obtained from 20 AVMA-accredited colleges of veterinary medicine. Email messages were sent in August 2015 to the 49 worldwide AVMA-accredited veterinary schools requesting a copy of their textbook lists, library reserve lists, or both. Librarians from 20 schools responded (40.8% response rate) and supplied the most recent lists that they had available.

Published veterinary bibliographies, representing the input of veterinary librarians, were limited to those published within the past fifteen years. These included the “Animal Health and Veterinary Science” chapter in *Using the Agricultural, Environmental, and Food Literature* [26], “Veterinary Medicine Books Recommended for Academic Libraries” [12], the “Veterinary Medicine” chapter of *Medical Library Association’s Master Guide to Authoritative Information Resources in the Health Sciences* [34], and the Veterinary Medicine section of Doody’s Core Titles in the Health Sciences [10].

### Data manipulation

Each source list was entered into an Excel spreadsheet. The bibliographic information was normalized into a common format. These lists were then combined into a single spreadsheet with each citation retaining an indication of its source list. After correcting erroneous bibliographic information, duplicate titles and alternate editions were merged into a single entry for each monograph. Each entry included the bibliographic information for the most recent edition of the item and indications of all source lists on which it occurred.

### Category lists

To generate core and essential lists of titles, the authors initially ranked the titles by how frequently they occurred. However, this list overrepresented titles that recurred in categories with many component lists (such as the AVMA lists), while titles that were found in less robust categories (such as citation data) were not well represented. To reduce this bias, lists of titles that occurred on the most source lists per category were generated. These were determined by looking for natural breaks in the data that would result in approximately 100 titles for each category [35]. This resulted in 102 titles that were on at least 2 cited book lists, 97 titles that were on at least 3 AVMA reading lists, 78 titles that were on at least 2 librarian bibliographies, and 91 titles that were on at least 8 textbook or library reserve lists. The overall title list was ranked according to the number of category lists on which each title appeared.

### Core and essential lists

As the natural break point in the ranked list that relied on category lists alone resulted in a list of only 85 titles that were on at least 2 category lists, an additional component was included to generate a more robust title list. In addition to the top titles for each category, the most frequently occurring titles overall were the 117 titles that were on least 11 source lists. The 4 category lists and the overall most common lists were weighted equally in developing the core and essential lists. Titles were sorted by the count of the total number of occurrences on the category and overall lists. The core list was generated by limiting to the 122 titles that were on 2 or more category and overall lists. The essential list was the subset of the core list consisting of 33 titles that occurred on 4 or more category and overall lists.

### Scope

The most recent edition of each included book title was listed in the core and essential lists as determined by searching publisher websites and WorldCat.Org. Forthcoming edition information was not included. Judgment was not made about whether previous, classic editions were more desirable. Titles were limited to English-language books with an emphasis on recent materials. All materials were available in print and sometimes additional formats at the time of compilation. This list does not include self-study or examination study materials. Some core lists provide pricing. The present lists do not include pricing because the current nature of pricing packages and consortial agreements makes meaningful pricing difficult.

## RESULTS

The essential list of 33 titles is provided in supplemental Appendix A. The core list of 122 titles is provided in supplemental Appendix B.

## DISCUSSION

A challenge in veterinary medicine core monograph lists is the use of monographs for multiple purposes and a blurring of lines between subcollections: reference, class reserve, textbooks, and clinical professional collections. The benefits of the lists compiled by the present study are their currency and their data-driven methodology. The main advantages of this methodology are its ease of use and adaptability to subjects beyond veterinary medicine. These lists are not designed to be, nor should they be taken as, comprehensive. Local needs must be considered and drive collections.

### Limitations of sources

The lists are limited to English-language, recent materials and may miss classics. The subject coverage is uneven and does not include standard references (e.g., dictionaries, protocols, directories, encyclopedias). Additionally, the lists miss newer areas of interest or emphasis in veterinary education and nonclinical and soft skills (e.g., communication, ethics, business, informatics, information literacy, veterinary medical translation) that do not appear frequently in the source lists. Additionally, the lists may be geographically limited; the RVSO reading lists had a North American focus, but the curricular contributions were worldwide.

### Citation data

Citation data are not as readily available for monographs as for journal articles. In addition to the three monographic citation indexes used, Google Scholar indexes veterinary monographic citation data. However, Google Scholar citation data were not used because search results are difficult to export, the search results cannot be replicated, and the source data are not transparent and can change.

A limitation of searching WOS and BIOSIS by “veterinary” subject is that the results are focused on veterinary science, not clinical veterinary medicine [36]. This was acceptable because the out-of-scope items would not be represented in the final essential and core lists. Repetition of this method would need to take into consideration that WOS was transitioning from Thomson Reuters to Clarivate Analytics when the data were searched and downloaded, and would need to account for any subsequent changes in coverage or interface functionality.

In addition to the limitations above, a limitation of searching Scopus for “veterinary” as a keyword is that not all works on veterinary medicine will include the term “veterinary” in the title, abstract, or keywords. Again, these limitations were acceptable because the citations for out-of-scope items would not be in other source lists and, therefore, not represented in the final compilations.

### American Veterinary Medical Association list data

The AVMA BSCE list is the only source focusing on basic clinical science titles. These titles might not be represented on core and essential lists because other sources focus on veterinary medicine rather than the basic clinical sciences.

Data from the AVMA RVSO lists are limited because not all veterinary specialties are represented: AVMA has twenty-two RVSOs representing forty-one specialties [37], but only thirty-five board examinations have reading lists. Additionally, not all specialties have examination reading lists, the lists are inconsistent with regard to depth of subject coverage, and the lists are inconsistent with regard to source materials (e.g., articles, monographs, guidelines, other materials).

### Published veterinary bibliographies

The four previously published veterinary book lists used as source lists for this study rely on expert opinion and, although most provide a methodology, they lack detailed transparency [10, 12, 26, 34].

### Use of data

There is no list of books or journals that AVMA requires for their accreditation process for veterinary schools. Established veterinary school libraries will very likely already cover these topics and include these, or similar, items based on their local needs and instructor preferences. The lists are not intended to specifically support laboratory animal research programs or institutional animal care and use committee or animal use protocol searches and permissions that accompany them.

With the preponderance of modern collection development focusing on packages and approval plans rather than individual selection, these lists will be most useful for libraries that do not focus primarily on veterinary collections and require narrowly focused subcollections to support local needs. In particular, these lists may be useful for collection development or benchmarking to support medical schools, allied health programs, pre-veterinary undergraduate curricula, veterinary research departments, new veterinary programs, and veterinary technician or nursing programs. As the lists have a North American geographical bias and an AVMA educational bias, they may be of particular use to programs outside those boundaries for benchmarking. In addition to specific institutions and programs, these lists are useful to those who are new to the subject or the collection development process.

## SUPPLEMENTAL FILES

Appendix A Essential list of veterinary monographs

Appendix B Core list of veterinary monographs
