# Supplementary material for: Essential and core books for veterinary medicine
Source: J Med Libr Assoc. 2018 Jul 1;106(3):304–10. doi: 10.5195/jmla.2018.391 (PMC6013128; doi:10.5195/jmla.2018.391)
Supplement: Appendix A [file jmla-106-304-s001.pdf]

JMLA Journal of the Medical Library Association

SC

Supplemental content to
J Med Libr Assoc. Jul;106(3):dx.doi.org/10.5195/jmla.2018.391
www.jmla.mlanet.org
© Moberly, Page 2018

Essential and core books for veterinary medicine

Heather K. Moberly, MSLS, AHIP; Jessica R. Page, MS, MLIS, AHIP

APPENDIX A

Essential list of veterinary monographs

1. Feldman EC, Nelson RW, Reusch C, Scott-Moncrieff JCR, Behred EN. Canine and feline endocrinology. 4th ed. St. Louis, MO: Elsevier Saunders; 2015.
2. Klein BG, Klein BG, eds. Cunningham's textbook of veterinary physiology. 5th ed. St. Louis, MO: Elsevier Saunders; 2013.
3. Youngquist RS, Threlfall WR, eds. Current therapy in large animal theriogenology. 2nd ed. St. Louis, MO: Saunders Elsevier; 2007.
4. Swayne DE, ed. Diseases of poultry. 13th ed. Ames, IA: John Wiley & Sons; 2013.
5. Zimmerman JJ, Karriker LA, Ramirez A, Schwartz KJ, Stevenson GW, eds. Diseases of swine. 10th ed. Ames, IA: Wiley-Blackwell; 2012.
6. Latimer KS, Duncan JR, eds. Duncan & Prasse's veterinary laboratory medicine: clinical pathology. 5th ed. Ames, IA: Wiley-Blackwell; 2011.
7. Singh B, Dyce KM, eds. Dyce, Sack and Wensing's textbook of veterinary anatomy. 5th ed. St. Louis, MO: Elsevier; 2018.
8. Reed SM, Bayly WM, Sellon DC, eds. Equine internal medicine. 4th ed. St. Louis, MO: Elsevier; 2018.
9. Auer JA, Stick JA, eds. Equine surgery. 4th ed. St. Louis, MO: Elsevier Saunders; 2012.
10. DiBartola SP, ed. Fluid, electrolyte, and acid-base disorders in small animal practice. 4th ed. St. Louis, MO: Elsevier Saunders; 2012.
11. Miller RE, Fowler ME, eds. Fowler's zoo and wild animal medicine. Volume 8 [i.e., 8th ed.]. St. Louis, MO: Elsevier; 2015.*
12. Muir W, Hubbell JAE, Bednarski RM, Lerche P. Handbook of veterinary anesthesia. 5th ed. St. Louis, MO: Elsevier Mosby; 2013.
13. Lorenz MD, Coates JR, Kent M. Handbook of veterinary neurology. 5th ed. St. Louis, MO: Elsevier Saunders; 2011.
14. Greene CE, ed. Infectious diseases of the dog and cat. 4th ed. St. Louis, MO: Elsevier Saunders; 2012.
15. Bonagura J, Twedt DC, eds. Kirk's current veterinary therapy (series). 1st–15th ed. Philadelphia, PA: Elsevier Saunders; 2014.†
16. Smith BP, ed. Large animal internal medicine. 5th ed. St. Louis, MO: Elsevier Mosby; 2015.
17. Smith FWK, Tilley LP, Oyama MA, Sleeper MM, eds. Manual of canine and feline cardiology. 5th ed. St. Louis, MO: Elsevier; 2016.
18. Aiello SE, Moses MA, eds. Merck veterinary manual. 11th ed. Kenilworth, NJ: Merck; 2016.
19. Miller WH, Griffin CE, Campbell KL. Muller & Kirk's small animal dermatology. 7th ed. St. Louis, MO: Elsevier Mosby; 2013.

---

* *Fowler's Zoo Animal Medicine* follows a full volume, updates, full volume publication pattern. Full volumes are titled *Fowler's Zoo and Wild Animal Medicine* (edition number). Updates are published under the title *Fowler's Zoo and Wild Animal Medicine: Current Therapy* (edition number). After a few updates, another full volume will incorporate the updates. All edition numbers are continuous. Selectors are advised to purchase the most recent full volume and use local need to determine if intermediate current therapy volumes are needed.

† *Kirk's Current Therapy* carries an edition designation; however, each volume contains new information, not an update of the previous edition.

Supplemental content to
J Med Libr Assoc. Jul;106(3):dx.doi.org/10.5195/jmla.2018.391
www.jmla.mlanet.org
© Moberly, Page 2018

20. Zachary JF, ed. Pathologic basis of veterinary disease. 6th ed. St. Louis, MO: Elsevier; 2017.
21. Plumb DC. Plumb's veterinary drug handbook. 8th ed. Stockholm, WI: PharmaVet; 2015.
22. Boothe DM, Boothe DM, eds. Small animal clinical pharmacology and therapeutics. 2nd ed. St. Louis, MO: Elsevier Saunders; 2012.
23. Nelson RW, Couto CG, eds. Small animal internal medicine. 5th ed. St. Louis, MO: Elsevier Mosby; 2014.
24. Fossum TW, ed. Small animal surgery. 4th ed. St. Louis, MO: Elsevier Mosby; 2013.
25. Slatter DH, ed. Textbook of small animal surgery. 3rd ed. Philadelphia, PA: Saunders; 2003.
26. Ettinger SJ, Feldman EC, Côté E, eds. Textbook of veterinary internal medicine: diseases of the dog and the cat. 8th ed. St. Louis, MO: Elsevier; 2017.
27. Grimm KA, Lamont LA, Tranquilli WJ, Greene SA, Robertson SA, eds. Veterinary anesthesia and analgesia: the fifth edition of Lumb and Jones. 5th ed. Ames, IA: John Wiley & Sons; 2015.
28. Tizard IR. Veterinary immunology: an introduction. 9th ed. St. Louis, MO: Elsevier; 2013.
29. Constable PD, Hinchcliff KW, Done SH, Gruenberg W. Veterinary medicine: a textbook of the diseases of cattle, horses, sheep, pigs, and goats. 11th ed. St. Louis, MO: Elsevier; 2017.
30. Gelatt KN, Gilger BC, Kern TJ, eds. Veterinary ophthalmology. 5th ed. Ames, IA: Wiley-Blackwell; 2013.
31. Riviere JE, Papich MG, eds. Veterinary pharmacology and therapeutics. 9th ed. Ames, IA: Wiley-Blackwell; 2009.
32. Gupta RC, ed. Veterinary toxicology: basic and clinical principles. 2nd ed. London, UK: Elsevier; 2012.
33. Withrow SJ, Vail DM, Page RL, eds. Withrow & MacEwen's small animal clinical oncology. 5th ed. St. Louis, MO: Elsevier Saunders; 2013.
