# Supplementary material for: Essential and core books for veterinary medicine
Source: J Med Libr Assoc. 2018 Jul 1;106(3):304–10. doi: 10.5195/jmla.2018.391 (PMC6013128; doi:10.5195/jmla.2018.391)
Supplement: Appendix B [file jmla-106-304-s002.pdf]

JMLA Journal of the Medical Library Association

SC

Supplemental content to
J Med Libr Assoc. Jul;106(3):dx.doi.org/10.5195/jmla.2018.391
www.jmla.mlanet.org
© Moberly, Page 2018

Essential and core books for veterinary medicine

Heather K. Moberly, MSLS, AHIP; Jessica R. Page, MS, MLIS, AHIP

APPENDIX B

Core list of veterinary monographs

1. Baxter GM, Adams OR, eds. Adams and Stashak's lameness in horses. 6th ed. Ames, IA: Wiley-Blackwell; 2011.
2. Wright KM, Whitaker BR, eds. Amphibian medicine and captive husbandry. Malabar, FL: Krieger Publishing Company; 2001.
3. Fascetti AJ, Delaney SJ, eds. Applied veterinary clinical nutrition. Ames, IA: Wiley-Blackwell; 2012.
4. Little S, ed. August's consultations in feline internal medicine. Vol. 7 [i.e., 7th ed.]. St Louis, MO: Elsevier; 2016.
5. Samour J, ed. Avian medicine. 3rd ed. St. Louis, MO: Elsevier; 2016.
6. Ritchie BW, ed. Avian medicine: principles and application. Delray Beach, FL: HBD International; 1999.
7. Landsberg GM, Hunthausen WL, Ackerman LJ. Behavior problems of the dog and cat. 3rd ed. Philadelphia, PA: Saunders Elsevier; 2013.
8. Fox JG, Marini RP, eds. Biology and diseases of the ferret. 3rd ed. Ames, IA: Wiley-Blackwell; 2014.
9. Tilley LP, Smith Jr. FWK, eds. Blackwell's five-minute veterinary consult: canine and feline. 6th ed. Ames, IA: John Wiley and Sons; 2016.
10. De Camp CE, Johnston SA, Déjardin LM, Schaefer SL, Brinker WO, Piermattei DL, Flo GL. Brinker, Piermattei and Flo's handbook of small animal orthopedics and fracture repair. 5th ed. St. Louis, MO: Elsevier; 2016.
11. Tutt C, Deeprose J, Crossley D, British Small Animal Veterinary Association, eds. BSAVA manual of canine and feline dentistry. 3rd ed. Quedgeley, Gloucester, UK: British Small Animal Veterinary Association; 2007. (BSAVA manuals.)
12. Feldman EC, Nelson RW, Reusch C, Scott-Moncrieff JCR, Behred EN. Canine and feline endocrinology. 4th ed. St. Louis, MO: Elsevier Saunders; 2015.
13. Sykes JE, ed. Canine and feline infectious diseases. St. Louis, MO: Elsevier Saunders; 2014.
14. Johnston SD, Root Kustritz MV, Olson PS. Canine and feline theriogenology. 1st ed. Philadelphia, PA: W. B. Saunders Company; 2001.
15. Ware WA. Cardiovascular disease in small animal medicine. 3rd impression, 2nd copyright. London, UK: Manson Publishing; 2011.
16. O'Malley B. Clinical anatomy and physiology of exotic species: structure and function of mammals, birds, reptiles, and amphibians. Philadelphia, PA: Elsevier Saunders; 2005.
17. Harrison GJ, Lightfoot TL, eds. Clinical avian medicine. Palm Beach, FL: Spix Publishing; 2006.
18. Kaneko JJ, Harvey JW, Bruss ML, eds. Clinical biochemistry of domestic animals. 6th ed. Burlington, MA: Academic Press Elsevier; 2008.
19. Butler JA, Colles CM, Dyson SJ, Kold SE, Poulos PW. Clinical radiology of the horse. 4th ed. Ames, IA: John Wiley & Sons; 2017.
20. Plumlee KH, ed. Clinical veterinary toxicology. St. Louis, MO: Mosby; 2004.
21. Klein BG, Klein BG, eds. Cunningham's textbook of veterinary physiology. 5th ed. St. Louis, MO: Elsevier Saunders; 2013.

SC
Supplemental content to
J Med Libr Assoc. Jul;106(3):dx.doi.org/10.5195/jmla.2018.391
www.jmla.mlanet.org
© Moberly, Page 2018

22. Bojrab MJ, Waldron DR, Toombs JP, eds. Current techniques in small animal surgery. 5th ed. Jackson, WY: Teton NewMedia; 2014.
23. Youngquist RS, Threlfall WR, eds. Current therapy in large animal theriogenology. 2nd ed. St. Louis, MO: Saunders Elsevier; 2007.
24. Mader DR, Divers SJ, eds. Current therapy in reptile medicine and surgery. St. Louis, MO: Elsevier Saunders; 2014.
25. Anderson DE, Rings DM, eds. Current veterinary therapy: food animal practice. 5th ed. St. Louis, MO: Saunders Elsevier; 2009.
26. Eurell JAC, Frappier BF, Dellmann HD, eds. Dellman's textbook of veterinary histology. 6th ed. Ames, IA: Blackwell Publishing; 2006.
27. Ross MW, Dyson SJ, eds. Diagnosis and management of lameness in the horse. 2nd ed. St. Louis, MO: Elsevier Saunders; 2011.
28. Swayne DE, ed. Diseases of poultry. 13th ed. Ames, IA: John Wiley & Sons; 2013.
29. Aitken ID, ed. Diseases of sheep. 4th ed. Oxford: Blackwell Science; 2007.
30. Zimmerman JJ, Karriker LA, Ramirez A, Schwartz KJ, Stevenson GW, eds. Diseases of swine. 10th ed. Ames, IA: Wiley-Blackwell; 2012.
31. Houpt KA. Domestic animal behavior for veterinarians and animal scientists. 5th ed. Ames, IA: Wiley-Blackwell; 2011.
32. Turner DC, Bateson PPG, eds. Domestic cat: the biology of its behaviour. 3rd ed. New York, NY: Cambridge University Press; 2014.
33. Reece WO, Erickson HH, Goff JP, Uemura EE, eds. Dukes' physiology of domestic animals. 13th ed. Ames, IA: Wiley Blackwell; 2015.
34. Latimer KS, Duncan JR, eds. Duncan & Prasse's veterinary laboratory medicine: clinical pathology. 5th ed. Ames, IA: Wiley-Blackwell; 2011.
35. Singh B, Dyce KM, eds. Dyce, Sack and Wensing's textbook of veterinary anatomy. 5th ed. St. Louis, MO: Elsevier; 2018.
36. Scott DW, Miller Jr. WH. Equine dermatology. 2nd ed. Maryland Heights, MO: Elsevier Saunders; 2011.
37. Sellon DC, Long MT, eds. Equine infectious diseases. 2nd ed. St. Louis, MO: Saunders Elsevier; 2014.
38. Reed SM, Bayly WM, Sellon DC, eds. Equine internal medicine. 4th ed. St. Louis, MO: Elsevier; 2018.
39. Gilger BC, Gilger BC, eds. Equine ophthalmology. 3rd ed. Ames, IA: John Wiley & Sons; 2017.
40. McKinnon AO, Squires EL, Vaala WE, Varner DD, eds. Equine reproduction. 2nd ed. Ames, IA: Wiley-Blackwell; 2011.
41. Auer JA, Stick JA, eds. Equine surgery. 4th ed. St. Louis, MO: Elsevier Saunders; 2012.
42. Gelatt KN, ed. Essentials of veterinary ophthalmology. 3rd ed. Ames, IA: John Wiley & Sons; 2014.
43. Fubini SL, Ducharme NG, eds. Farm animal surgery. 2nd ed. St. Louis, MO: Elsevier; 2017.
44. MacLachlan NJ, Dubovi EJ, Fenner F, eds. Fenner's veterinary virology. 5th ed. Amsterdam, The Netherlands: Elsevier/Academic Press; 2017.
45. Quesenberry KE, Carpenter JW, eds. Ferrets, rabbits, and rodents: clinical medicine and surgery. 3rd ed. St. Louis, MO: Elsevier Saunders; 2012.
46. DiBartola SP, ed. Fluid, electrolyte, and acid-base disorders in small animal practice. 4th ed. St. Louis, MO: Elsevier Saunders; 2012.

SC
Supplemental content to
J Med Libr Assoc. Jul;106(3):dx.doi.org/10.5195/jmla.2018.391
www.jmla.mlanet.org
© Moberly, Page 2018

47. Miller RE, Fowler ME, eds. Fowler's zoo and wild animal medicine. Vol. 8 [i.e., 8th ed.]. St. Louis, MO: Elsevier; 2015.*
48. Fowler ME. Fowler's zoo and wild animal medicine: current therapy. Vol. 7 [i.e., 7th ed.]. Miller, Fowler, ed. St. Louis, MO: Elsevier Saunders; 2012.*
49. Bagley RS. Fundamentals of veterinary clinical neurology. 1st ed. Ames, IA: Blackwell Publishing; 2005.
50. Stockham SL, Scott MA. Fundamentals of veterinary clinical pathology. 2nd ed. Ames, IA: Blackwell; 2008.
51. Bowman DD, Georgi JR. Georgis' parasitology for veterinarians. 10th ed. St. Louis, MO: Elsevier Saunders; 2014.
52. Smith MC, Sherman DM. Goat medicine. 2nd ed. Ames, IA: Wiley-Blackwell; 2009.
53. Brunton LL, Chabner B, Knollmann BC, Knollmann BC, eds. Goodman & Gilman's pharmacological basis of therapeutics. 12th ed. New York, NY: McGraw-Hill; 2011.
54. Evans H, DeLahunta A. Guide to the dissection of the dog. 8th ed. St. Louis, MO: Elsevier; 2017.
55. Hall JE. Guyton and Hall textbook of medical physiology. 13th ed. Philadelphia, PA: Elsevier; 2016.
56. Morgan RV, ed. Handbook of small animal practice. 5th ed. St. Louis, MO: Saunders Elsevier; 2008.
57. Muir W, Hubbell JAE, Bednarski RM, Lerche P. Handbook of veterinary anesthesia. 5th ed. St. Louis, MO: Elsevier Mosby; 2013.
58. Lorenz MD, Coates JR, Kent M. Handbook of veterinary neurology. 5th ed. St. Louis, MO: Elsevier Saunders; 2011.
59. Gaynor JS, Muir III WW, eds. Handbook of veterinary pain management. 3rd ed. St. Louis, MO: Elsevier; 2015.
60. Radostits OM. Herd health: food animal production medicine. 3rd ed. Philadelphia, PA: Saunders; 2001.
61. Jacobson ER, ed. Infectious diseases and pathology of reptiles: color atlas and text. Boca Raton, FL: CRC Press/Taylor & Francis Group; 2007.
62. Greene CE, ed. Infectious diseases of the dog and cat. 4th ed. St. Louis, MO: Elsevier Saunders; 2012.
63. Maxie MG, ed. Jubb, Kennedy, and Palmer's pathology of domestic animals. 6th ed. St. Louis, MO: Elsevier; 2016.
64. Bonagura J, Twedt DC, eds. Kirk's current veterinary therapy (series). 1st–15th ed. Philadelphia, PA: Elsevier Saunders; 2014.†
65. Fox JG, Anderson LC, Otto GM, Pritchett-Corning KR, Whary MT, American College of Laboratory Animal Medicine, eds. Laboratory animal medicine. 3rd ed. London, UK: Academic Press Elsevier; 2015. (American College of Laboratory Animal Medicine series.)
66. Suckow MA, Stevens KA, Wilson RP, eds. Laboratory rabbit, guinea pig, hamster, and other rodents. 1st ed. London, UK: Academic Press Elsevier; 2012. (American College of Laboratory Animal Medicine series.)
67. Smith BP, ed. Large animal internal medicine. 5th ed. St. Louis, MO: Elsevier Mosby; 2015.

---

* *Fowler's Zoo Animal Medicine* follows a full volume, updates, full volume publication pattern. Full volumes are titled *Fowler's Zoo and Wild Animal Medicine* (edition number). Updates are published under the title *Fowler's Zoo and Wild Animal Medicine: Current Therapy* (edition number). After a few updates, another full volume will incorporate the updates. All edition numbers are continuous. Selectors are advised to purchase the most recent full volume and use local need to determine if intermediate current therapy volumes are needed.

† *Kirk's Current Therapy* carries an edition designation; however, each volume contains new information not an update of the previous edition.

Supplemental content to
J Med Libr Assoc. Jul;106(3):dx.doi.org/10.5195/jmla.2018.391
www.jmla.mlanet.org
© Moberly, Page 2018

68. Mayhew IGJ. Large animal neurology. 2nd ed. Chichester, West Sussex, UK: Wiley-Blackwell; 2009.
69. Smith FWK, Tilley LP, Oyama MA, Sleeper MM, eds. Manual of canine and feline cardiology. 5th ed. St. Louis, MO: Elsevier; 2016.
70. Brinsko SP, Blanchard TL, Varner DD, Schumacher J, Love CC, Hinrichs K, Hartman D, eds. Manual of equine reproduction. 3rd ed. Maryland Heights, MO: Mosby Elsevier; 2011.
71. Luescher AU, ed. Manual of parrot behavior. 1st ed. Ames, IA: Blackwell Publishing; 2006.
72. Fowler ME, Bravo PW. Medicine and surgery of camelids. 3rd ed. Ames, IA: Wiley-Blackwell; 2010.
73. Aiello SE, Moses MA, eds. Merck veterinary manual. 11th ed. Kenilworth, NJ: Merck; 2016.
74. Evans HE, de Lahunta A. Miller's anatomy of the dog. 4th ed. St. Louis, MO: Elsevier Saunders; 2013.
75. Miller WH, Griffin CE, Campbell KL. Muller & Kirk's small animal dermatology. 7th ed. St. Louis, MO: Elsevier Mosby; 2013.
76. Bartges J, Polzin DJ, eds. Nephrology and urology of small animals. Ames, IA: Wiley-Blackwell; 2011.
77. Zachary JF, ed. Pathologic basis of veterinary disease. 6th ed. St. Louis, MO: Elsevier; 2017.
78. Senger PL. Pathways to pregnancy & parturition. 3rd ed. Redmond, OR: Current Conceptions; 2012.
79. Johnson KA. Piermattei's atlas of surgical approaches to the bones and joints of the dog and cat. 5th ed. St. Louis, MO: Elsevier Saunders; 2014.
80. Plumb DC. Plumb's veterinary drug handbook. 8th ed. Stockholm, WI: PharmaVet; 2015.
81. Dewey CW, da Costa RC, eds. Practical guide to canine and feline neurology. 3rd ed. Ames, IA: Wiley-Blackwell; 2016.
82. Divers TJ, Peek SF. Rebhun's diseases of dairy cattle. 2nd ed. St. Louis, MO: Saunders Elsevier; 2008.
83. Mader DR, ed. Reptile medicine and surgery. 2nd ed. St. Louis, MO: Saunders Elsevier; 2006.
84. Kumar V, Abbas AK, Aster JC, eds. Robbins and Cotran pathologic basis of disease. 9th ed. Philadelphia, PA: Elsevier Saunders; 2015.
85. Sprayberry K, Robinson NE, eds. Robinson's current therapy in equine medicine. 7th ed. St. Louis, MO: Elsevier Saunders; 2015.
86. Birchard SJ, Sherding RG, eds. Saunders manual of small animal practice. 3rd ed. St. Louis, MO: Saunders Elsevier; 2006.
87. Weiss DJ, Wardrop KJ, Schalm OW, eds. Schalm's veterinary hematology. 6th ed. Ames, IA: Wiley-Blackwell; 2010.
88. Pugh DG, Baird AN, eds. Sheep and goat medicine. 2nd ed. Maryland Heights, MO: Elsevier/Saunders; 2012.
89. Gross TL, Ihrke PJ, Walder EJ, Affolter VK, eds. Skin diseases of the dog and cat: clinical and histopathologic diagnosis. 2nd ed. Ames, IA: Blackwell Science; 2005.
90. Maggs DJ, Miller PE, Ofri R, Slatter DH. Slatter's fundamentals of veterinary ophthalmology. 6th ed. St. Louis, MO: Elsevier; 2018.
91. Kittleson MD, Kienle RD. Small animal cardiovascular medicine. St. Louis, MO: Mosby; 1998.
92. Willard MD, Tvedten H, eds. Small animal clinical diagnosis by laboratory methods. 5th ed. St. Louis, MO: Elsevier Saunders; 2012.
93. Hand M, Thatcher LD, Remillard RL, Roudebush P, Novotny BJ, eds. Small animal clinical nutrition. 5th ed. Topeka, KS: Mark Morris Institute; 2010.

Supplemental content to
J Med Libr Assoc. Jul;106(3):dx.doi.org/10.5195/jmla.2018.391
www.jmla.mlanet.org
© Moberly, Page 2018

94. Boothe DM, Boothe DM, eds. Small animal clinical pharmacology and therapeutics. 2nd ed. St. Louis, MO: Elsevier Saunders; 2012.
95. Silverstein DC, Hopper K, eds. Small animal critical care medicine. 2nd ed. St. Louis, MO: Elsevier Saunders; 2015.
96. Hnilica KA, Patterson AP. Small animal dermatology: a color atlas and therapeutic guide. 4th ed. St. Louis, MO: Elsevier; 2017.
97. Nelson RW, Couto CG, eds. Small animal internal medicine. 5th ed. St. Louis, MO: Elsevier Mosby; 2014.
98. Peterson ME, Kutzler MA, eds. Small animal pediatrics: the first 12 months of life. St. Louis, MO: Elsevier Saunders; 2011.
99. Sharp NJH, Wheeler SJ. Small animal spinal disorders: diagnosis and surgery. 2nd ed. Edinburgh, UK: Elsevier Mosby; 2005.
100. Fossum TW, ed. Small animal surgery. 4th ed. St. Louis, MO: Elsevier Mosby; 2013.
101. Peterson ME, Talcott PA, Peterson ME, eds. Small animal toxicology. 3rd ed. St. Louis, MO: Elsevier Saunders; 2013.
102. Slatter DH, ed. Textbook of small animal surgery. 3rd ed. Philadelphia, PA: Saunders; 2003.
103. Thrall DE, ed. Textbook of veterinary diagnostic radiology. 6th ed. St. Louis, MO: Elsevier Saunders; 2013.
104. Ettinger SJ, Feldman EC, Côté E, eds. Textbook of veterinary internal medicine: diseases of the dog and the cat. 8th ed. St. Louis, MO: Elsevier; 2017.
105. Meuten DJ, ed. Tumors in domestic animals. 5th ed. Ames, IA: John Wiley & Sons; 2017.
106. Grimm KA, Lamont LA, Tranquilli WJ, Greene SA, Robertson SA, eds. Veterinary anesthesia and analgesia: the fifth edition of Lumb and Jones. 5th ed. Ames, IA: John Wiley & Sons; 2015.
107. Smith RD. Veterinary clinical epidemiology. 3rd ed. Boca Raton, FL: CRC Press/Taylor & Francis Group; 2006.
108. Zajac A, Conboy GA. Veterinary clinical parasitology. 8th ed. Ames, IA: Wiley-Blackwell; 2012.
109. Thrall MA, ed. Veterinary hematology and clinical chemistry. 2nd ed. Ames, IA: Wiley-Blackwell; 2012.
110. Tizard IR. Veterinary immunology: an introduction. 9th ed. St. Louis, MO: Elsevier; 2013.
111. Constable PD, Hinchcliff KW, Done SH, Gruenberg W. Veterinary medicine: a textbook of the diseases of cattle, horses, sheep, pigs, and goats. 11th ed. St. Louis, MO: Elsevier; 2017.
112. McVey DS, Kennedy M, Chengappa MM, eds. Veterinary microbiology. 3rd ed. Ames, IA: Wiley-Blackwell; 2013.
113. Quinn PJ, Markey OK, Leonard FL, FitzPatrick ES, Fanning S, Hartigen PJ, eds. Veterinary microbiology and microbial disease. 2nd ed. Oxford, UK: Wiley-Blackwell; 2011.
114. DeLahunta A, Glass E, Kent M. Veterinary neuroanatomy and clinical neurology. 4th ed. St. Louis, MO: Elsevier Saunders; 2015.
115. Gelatt KN, Gilger BC, Kern TJ, eds. Veterinary ophthalmology. 5th ed. Ames, IA: Wiley-Blackwell; 2013.
116. Taylor MA, Coop RL, Wall R. Veterinary parasitology. 4th ed. Ames, IA: Wiley-Blackwell; 2016.
117. Riviere JE, Papich MG, eds. Veterinary pharmacology and therapeutics. 9th ed. Ames, IA: Wiley-Blackwell; 2009.

SC
Supplemental content to
J Med Libr Assoc. Jul;106(3):dx.doi.org/10.5195/jmla.2018.391
www.jmla.mlanet.org
© Moberly, Page 2018

118. Johnston SA, Tobias KM, eds. Veterinary surgery: small animal. 2nd ed. St. Louis, MO: Elsevier; 2018.
119. Gupta RC, ed. Veterinary toxicology: basic and clinical principles. 2nd ed. London, UK: Elsevier; 2012.
120. West JB, Luks AM. West's respiratory physiology: the essentials. 10th ed. Philadelphia, PA: Wolters Kluwer; 2016.
121. Withrow SJ, Vail DM, Page RL, eds. Withrow & MacEwen's small animal clinical oncology. 5th ed. St. Louis, MO: Elsevier Saunders; 2013.
122. West G, Heard D, Caulkett N, eds. Zoo animal and wildlife immobilization and anesthesia. 2nd ed. Ames, IA: Wiley Blackwell; 2014.
